# A Home for Feeble-Minded Girls

**Published:** 1910-04-02

**Authors:** Margaret Brudenell-Bruce, Lucy C. F. Cavendish, William Chance, W. H. Dickinson, C. S. Loch, Constance B. Merser, George H. Savage

**Affiliations:** National Association for the Feeble-minded, Denison House, 296 Vauxhall Bridge Road, S.W.; National Association for the Feeble-minded, Denison House, 296 Vauxhall Bridge Road, S.W.; National Association for the Feeble-minded, Denison House, 296 Vauxhall Bridge Road, S.W.; National Association for the Feeble-minded, Denison House, 296 Vauxhall Bridge Road, S.W.; National Association for the Feeble-minded, Denison House, 296 Vauxhall Bridge Road, S.W.; National Association for the Feeble-minded, Denison House, 296 Vauxhall Bridge Road, S.W.; National Association for the Feeble-minded, Denison House, 296 Vauxhall Bridge Road, S.W.


### Editor's Letter-Box.

### A HOME FOR FEEBLE-MINDED GIRLS.

*To the Editor of* The Hospital.

Sir,—Every year newspapers teem with figures demonstrating the increase of feeble-mindedness, lunacy, and the crimes resulting from these causes; yet the public look on at this rising flood of degeneracy without attempting to avert it.

"If there is a positive increase in the numbers of the feeble-minded and of lunatics," says Professor Clifford Allbutt, "it is because we are doing our best to breed them." Is the nation becoming so effete that its individual members can do nothing to end such conditions? If so, it is not surprising that feeble-mindedness increases.

We appeal to all who can appreciate the seriousness of the problem to help us to grapple with it. Feeble-mindedness is largely hereditary, and it is therefore one of the most preventable of diseases, provided only that those afflicted, while being kindly treated and well cared for, can be kept apart from the world. For this reason, the Association has founded a farm colony of 170 acres near Tonbridge, where, by the care and control of the feeble-minded, one of the most subtle evils which has ever attacked national life is being successfully combated. The colony is named after the Princess Christian, and she will herself open it on June 3 next. Already there is established a home for lads of sixteen to twenty-three; these lads in some cases a source of danger to all around them, are now busily and happily employed in tending the live stock which kind sympathisers have already given, and in other colony work. So far a sum of £4,300 has been subscribed for the colony, but another £8,000 is yet required (1) to clear off the debt for the land purchased, (2) to build more homes, (3) to establish a school and homes for feeble-minded children of both sexes.

An earnest appeal is made for the immediate foundation of a home for young girls, who are in greater danger than all others. For this about £1,500 will be needed, and a great effort is being made to collect the sum by June 3, in order that the Princess Christian may lay the foundation stone. For every £100 we can add a named bed to the colony. Furniture, clothing, and farm stock will be gratefully accepted.

It is probably too much to hope that this scheme will appeal to the impulsive benevolence of the general public, but to those of wider knowledge, who can see its far-reaching issues, we make our request with much confidence. A medical specialist has said, "More nations have sunk to utter insignificance as the result of moral, intellectual, and physical degeneracy than by war, famine, or any other conditions." It is time that this menace to the British race was put an end to.

Will those who read this show their interest by sending a subscription or donation, marked either "Colony" or "General Fund," to the Secretary of the National Association for the Feeble-minded at Denison House, 296 Vauxhall Bridge Road, S.W.?

We are, your obedient servants,

Margaret Brudenell-Bruce.
Lucy C. F. Cavendish.
William Chance.
W. H. Dickinson.
C. S. Loch.
Constance B. Merser.
George H. Savage.

National Association for the Feeble-minded,
Denison House, 296 Vauxhall Bridge Road, S.W.,
March 8, 1910.

